# Enhanced preoperative education about continuous femoral nerve block with patient-controlled analgesia improves the analgesic effect for patients undergoing total knee arthroplasty and reduces the workload for ward nurses

**DOI:** 10.1186/s12871-019-0826-3

**Published:** 2019-08-14

**Authors:** Xiaona Lin, Yang Zhou, Hongcai Zheng, Jing Zhang, Xiaoxiao Wang, Kaixi Liu, Jun Wang, Xiangyang Guo, Zhengqian Li, Bin Han

**Affiliations:** 1 Department of Anesthesiology, Peking University Third Hospital, No. 49, North Garden Rd, Haidian District, Beijing, 100191 China; 2 Research Center of Clinical Epidemiology, Peking University Third Hospital, Beijing, 100191 China

**Keywords:** Total knee arthroplasty, Patient-controlled analgesia, Preoperative education, Nursing workload

## Abstract

**Background:**

Total knee arthroplasty (TKA) is the most common treatment for end-stage knee osteoarthritis and continuous femoral nerve block (CFNB) has become the gold standard for analgesia. But the lack of knowledge about CFNB and patient-controlled analgesia (PCA) often leads to inappropriate dose of medications and increased workload for ward nurses.

**Methods:**

After retrospectively registering to http://www.chictr.org.cn (ChiCTR1800018957), 60 patients undergoing unilateral TKA were randomly divided into groups A and B (*n* = 30 each). Patients in group B and their families received a nurse-led preoperative visit the day before surgery focusing on PCA educational pamphlets for postoperative pain management. Before returning to the ward, patients and their families in both groups received face-to-face PCA pump operation training. The usual postoperative follow-up was performed by nurse anesthetists for 2 consecutive days. Visual Analogue Scale (VAS) scores at rest and during movement, knowledge of the PCA evaluated by a ten-question questionnaire, knee flexion angles, and the number of PCA-related nurse calls were recorded.

**Results:**

The VAS scores at rest and during movement of the patients in group B were both significantly lower than in group A on postoperative days 1 and 2. The questionnaire scores of the patients in group B were much higher than those in group A on postoperative day 1, but not on day 2. Patients in the 2 groups had similar knee flexion on postoperative days 1 and 2. Patients in group B asked for assistance from the ward nurses with the PCA fewer times than those in group A, and the ward nurses were more satisfied with the analgesic protocol in group B.

**Conclusions:**

Enhanced preoperative education for CFNB with PCA can provide patients with a better grasp of postoperative pain management, improve the postoperative analgesic effect after TKA, and reduce the PCA-related workload for ward nurses.

**Trial registration:**

This study was retrospectively registered to ChiCTR (identifier: ChiCTR1800018957) on October 18, 2018.

**Electronic supplementary material:**

The online version of this article (10.1186/s12871-019-0826-3) contains supplementary material, which is available to authorized users.

## Background

Total knee arthroplasty (TKA) is the most common treatment for end-stage knee osteoarthritis [1, 2]. While the number of patients undergoing TKA is increasing annually with the aging global population, the management of postoperative pain has attracted much recent attention. It has been found that postoperative pain can directly affect early functional exercise and reduce the effectiveness of surgical treatment [3]. In recent years, continuous femoral nerve block (CFNB) has become the gold standard for analgesia after TKA [4]. However, the lack of knowledge by patients and their families about CFNB and patient-controlled analgesia (PCA) often leads to under-dosing or over-dosing of medications, a decrease in general analgesic efficacy, an increased workload for ward nurses, and an increased risk of falls [5]. While the importance of preoperative patient education has been emphasized, its role in improving postoperative analgesia and outcomes remains unclear. In this study, we explored whether preoperative CFNB education could improve postoperative analgesic efficacy; also, we evaluated whether preoperative CFNB education could reduce the PCA-related workload for ward nurses after TKA.

Accordingly, a prospective randomized clinical study was undertaken with the primary aim of evaluating the VAS on motion on POD1, and secondarily, to investigate the PCA-related workload for ward nurses postoperatively.

### Subjects and methods

This study was approved by the Ethics Committee of Peking University Third Hospital and recorded on October 18, 2018 to chictr.org.cn (ChiCTR1800018957).

In total, 60 patients undergoing unilateral TKA in our hospital at 08:00–12:00 between November 2016 and March 2018 were enrolled and randomly divided into 2 groups (group A and group B; *n* = 30 each) using a random number table. The inclusion criteria included: a) being American Society of Anesthesiologists (ASA) grade I - II; b) undergoing unilateral TKA (left or right); c) receiving combined spinal-epidural anesthesia. The exclusion criteria were: a) inability to complete VAS scoring; b) lower-extremity muscle strength below grade V; c) intraoperative intraarticular injection of analgesic drugs; d) history of previous TKA; e) contraindications to spinal manipulation or patient insisting on general anesthesia. Before returning to the ward after surgery, patients in group A and their families received unified face-to-face PCA pump operation training by a nurse anesthetist. In contrast, patients in group B and their families received a nurse-led preoperative visit the day before surgery focusing on a PCA educational pamphlet (see the Additional file 1) about postoperative pain management. After surgery, they watched the PCA demonstration with group A patients/families before returning to the ward.

CFNB was performed by the same anesthesiologist in the post anesthesia care unit (PACU) or operating room before or after TKA. Under ultrasound guidance combined with nerve stimulation, the femoral nerve catheter was placed, and a single dose of 0.5% ropivacaine (20 ml) was administered. All of the patients received combined spinal-epidural anesthesia by the same anesthesiologist, along with subarachnoid administration of 2–3 ml of 0.5% bupivacaine. The epidural catheter was routinely placed 4 cm ahead. During the operation, 1% lidocaine was added to maintain the anesthesia via the epidural catheter according to the operative time (3 ml 1% lidocaine for test doses and 5 ml added each time according to the effect to maintain the block level at about T10). The same TKA procedure was performed, during which the lower-extremity tourniquet was routinely applied. After the operation, the femoral nerve tube was connected to the PCA pump (500 ml of 0.2% ropivacaine, with background infusion rate 5 ml·h^− 1^, lockout interval 15 min, and the patient controlled bolus 2 ml). In addition to the continuous femoral nerve PCA, all of the patients were intravenously administered 40 mg of parecoxib sodium twice daily for two consecutive days.

### Data collection

After the patients returned to the ward, the ward surgeon routinely recorded the knee flexion angle, and the ward nurses recorded the number of PCA-related nurse calls. An appointed nurse anesthetist followed up the 2 groups for postoperative analgesia on the first and second days after the operation and carried out bedside education according to the specific PCA conditions. Meanwhile, the following data were collected: a) age, height, weight, ASA grade, timing and location of CFNB, time of operation, dosage of epidural drug, and level of postoperative anesthesia; b) VAS scores at rest and during movement on the first and second days after the operation; c) a ten-question questionnaire form (see the Additional file 1) on the first and second days after the operation, used to evaluate the patients’ knowledge about CFNB and PCA - the results of which both the nurse anesthetist and patients were blinded to before the end of the two surveys; and d) the satisfaction (satisfied, not satisfied, and indifferent) of the ward nurses to the PCA mode of CFNB.

### Statistical analysis

Power analysis was based on results of preliminary data comparing VAS score on motion on POD 1 between groups. In our preliminary study, 8 patients were recruited in each group. The medians and quartiles of VAS scores during movement were 4.5 ± 1 and 3 ± 1.75 in group A and B, respectively. Sample-size calculations showed that a Wilcoxon and Mann–Whitney U test with a type I error (two-sided) of α = 0.05 would have 95% power to detect the aforementioned difference in VAS scores on motion between the two groups if the sample size in each group was 24. To account for possible loss in the follow-up period, we enrolled 30 cases per group. All statistical analysis was performed with SPSS for Windows (version 14.0, SPSS, Chicago, IL). Data were tested for normal distribution using the Kolmogorov–Smirnov test. Continuous variables were presented using means and standard deviations (SD) or median and interquartile values, and analyzed using Student’s t test or the Wilcoxon-Mann-Whitney test, respectively. In the case of categorical variables, frequencies were used, and the count data were compared with a χ^2^ test. A *p* value of < 0.05 was regarded as statistically significant.

## Results

Follow-up and data collection were completed in all 60 patients. There were no significant differences in age, height, weight, and body mass index between the 2 groups (all *p* > 0.05). In both groups, 23 patients received CFNB in the PACU and 7 patients received it in the operating room (*p* > 0.05), and there was no significant difference between the groups in the timing of the CFNB. During intraoperative anesthesia management, 1 of 30 patients in Group A and 4 of 30 in Group B received additional epidural administration of 1% lidocaine (3.3% vs 13.3%, χ^2^ = 0.873, *p* = 0.350), the dosage of epidural lidocaine in Group A was 8 ml and the dosage in Group B was 5.5 (3, 8) ml. The anesthesia level immediate after surgery was maintained at about T10 in both groups, and there was no significant difference between the 2 groups (Table 1).

**Table 1:** Anthropometric subject characteristics

Variable	Group A (*n* = 30)	Group B (*n* = 30)	*P*-Values
Age	66.6 ± 6.5	66.5 ± 8.1	0.986
Gender (female/male)	22/8	27/3	0.095
Height (cm)	158.8 ± 7.5	159.0 ± 7.7	0.919
Weight (kg)	66.3 ± 8.8	67.6 ± 11.2	0.601
BMI (kg/m^2^)	26.4 ± 4.1	26.7 ± 4.0	0.756
ASA physical status (class I/II)	12/18	15/15	0.436
CFNB performance			
Side (left/right)	14/16	10/20	0.292
in PACU (pre−/post-op)	11/12	12/11	0.768
in OR (pre−/post-op)	6/1	7/0	1.000
Dosage of spinal bupivacaine (mg)	12.5 (12.5, 12.5)	12.5 (12.5, 12.5)	0.729
Upper thoracic sensory block level before surgery	10 (8, 10)	10 (8, 10)	0.629
Upper thoracic sensory block level immediately after surgery	10 (10, 10)	10 (10, 11)	0.564
Tourniquet application time (min)	62.1 ± 13.8	69.0 ± 14.7	0.349
Intraoperative blood loss (ml)	5 (5, 15)	10 (5, 20)	0.769
Duration of surgery (min)	74.3 ± 18.4	74.1 ± 18.1	0.972

Values are reported as mean ± SD or number of subjects. Non-normal data are expressed as median and interquartile values. *ASA* American Society of Anesthesiologists, *BMI* Body mass index, *CFNB* Continuous femoral nerve block

A 10-question questionnaire form was used to survey the patients on the first and the second postoperative days (Additional file 1). The results showed that the scores were significantly higher in group B than in group A on the first day (*p* = 0.01) (Table 2); however, the difference was not statistically significant on the second day (*p* = 0.24).

**Table 2:** Questionnaire scores for patients in two groups

Questionnaire scores	Group A (*n* = 30)	Group B (*n* = 30)	*P*-Values
POD 1	8.0 ± 1.4	9.4 ± 1.2	0.001
POD 2	9.8 ± 0.4	9.9 ± 0.3	0.235

Values are reported as mean ± SD. *POD* Postoperative day

The VAS scores at rest and during movement in group B were significantly lower than those in group A on both the first and the second day after surgery (*p* < 0.05). There was no significant difference in knee flexion angle between the groups 2 days after the operation (Table 3). In addition, no adverse events such as falls occurred in either group.

**Table 3:** Analgesic effect of CFNB in two groups

	Group A (*n* = 30)	Group B (*n* = 30)	*P*-Values
VAS at rest			
POD 1	1 (1,2)	0 (0,1)	<0.001
POD 2	0 (0,1)	0 (0,0)	0.005
VAS on motion			
POD 1	4 (4,5)	3 (2,5)	<0.001
POD 2	3 (3,4)	2 (2,2)	<0.001
Knee flexion			
POD 1	100 (90, 120)	100 (90, 120)	0.916
POD 2	108 (98, 120)	110 (99, 120)	0.811

Non-normal data are expressed as median and interquartile values. *CFNB* Continuous femoral nerve block, *VAS* Visual Analogue Scale

The number of PCA-related nurse calls was significantly greater in group A than in group B (*p* < 0.05). The survey about the satisfaction of ward nurses with regard to the PCA showed that 60% of the nurses responsible for patients in group A were satisfied, 6.7% were not satisfied, and 33% were indifferent. For group B, 93.3% of the nurses were satisfied and 6.7% were indifferent. The difference between the groups was statistically significant (*p* = 0.003) (Table 4).

**Table 4:** Nursing workload relief and satisfaction in the ward

	Group A (*n* = 30)	Group B (*n* = 30)	*P*-Values
Patients using PCA-related nurse call	15/15	7/23	0.032
Nursing satisfaction			
1	18 (60%)	28 (93.3%)	0.003
2	2 (6.7%)	0 (0%)	
3	10 (33%)	2 (6.7%)	

Values are reported as *n* and (percentages). *PCA* Patient-controlled analgesia

## Discussion

TKA is commonly performed to relieve joint pain, and to improve quality of life in patients who have end-stage osteoarthritis, but is also associated with severe pain after surgery and a high incidence of chronic pain [6]. CFNB is the most commonly used postoperative analgesic method for TKA, not only could provide effective postoperative analgesia but also could improve joint function and quality of life even at 1 month postoperatively [7]. However, how to operate the device and how to cope with postoperative pain by self-administering analgesic is a problem and the efficacy of PCA highly depends on the patients’ and their families’ knowledge and skills [8]. If patients receiving PCA correctly maintain their appropriate concentration, the effect could be good and should lead to the decrease in demand for drugs and ward nurses; however, this assumption related to the patient’s training has not been confirmed.

In the real-world clinical setting, the traditional preoperative oral education about PCA is often poorly understood by patients and their families, or quickly forgotten. Hekmatpou et al. found that about 80% of the information given to patients by their doctors was forgotten, and nearly half of the information remembered by patients was incorrect [9]. In our study, we developed a PCA educational pamphlet that focused on the knowledge of CFNB, PCA, and pain relief following TKA, with an attempt to intensify and improve preoperative PCA education. Our results suggested that the intensified education significantly improved the level of PCA-related knowledge in patients. However, in Yankova et al.’s study, although preoperative PCA education improved the use of the PCA device in patients, it showed no positive effect on postoperative analgesic efficacy (i.e., the VAS score) [10]. In contrast, our modified education model significantly lowered the VAS scores of patients at rest and during movement 2 days after surgery. Since the upper thoracic sensory block level immediately after surgery showed no significant differences between the groups, we followed up the patients on POD1 was 24 h postoperatively and no residual effect of central nerve blocks existed. Thus, we believe that the difference in postoperative analgesic efficacy might be explained by the preoperative education mode.

Since the VAS scores at rest and during movement on POD1 and POD2 in this research are quite similar, we doubt that the statistical significance may not make clinical difference. Actually, in the real-world clinical practice, no additional analgesics are provided when the VAS score is below 3. We hoped that readers could view these data objectively and carefully. From the perspective of clinical application, enhanced preoperative education about continuous femoral nerve block with PCA is still relatively limited in improving the postoperative analgesic effects.

PCA provides patients with a definite analgesic efficacy, allowing tailored self-management. However, while providing an analgesic effect, CFNB also remarkably weakens quadriceps femoris muscle strength, affects the early movement, and increases the risk of falls [11, 12]. Thacher et al. found that the first fall occurred 21 h (mean) after surgery [13]. In our study, fall precautions were highlighted in the pamphlet to improve postoperative safety. It was found that there were still 2 patients in group A that left the bed by themselves with walkers. One of them was a patient who was at high risk of falling less than 6 h after extubation. In group B, one patient became ambulatory with the assistance of his family members and medical staff 6 h after the catheter was removed. No falls occurred in our study, suggesting that the concentration and dosage of the analgesic drugs met the actual clinical needs.

Postoperative pain management is an ongoing challenge in surgical care and the nurses are very important participants [14]. The patients’ inadequate knowledge and erroneous beliefs may hamper the appropriate use of analgesics and increase the workload of ward nurses [15]. However, the impact of PCA on the workload of ward nurses remains unclear. King et al. found that patients would call ward nurses due to problems with the PCA pump and the analgesic effect, which may be sources of workload for ward nurses [16]. Our data showed that intensified preoperative education significantly decreased the number of nurse calls within 2 days after the operation and thus effectively reduced the workload for ward nurses. As a result, the satisfaction of ward nurses with regard to the PCA in these patients dramatically increased. Thus, the lower analgesia-related workload of ward nurses was associated with improved patient knowledge and skills about the PCA after the intensified education.

In summary, intensified preoperative analgesia education could be strengthened by the distribution of PCA educational materials, which allows patients and their families to have better knowledge and skills for pain relief after TKA, enhances their participation in postoperative rehabilitation, statistically improves the efficacy of postoperative analgesia, and definitely reduces workloads for ward nurses. Thus, preoperative PCA education can play an active role in pain management after TKA.

## Additional file

Additional file 1: This file shows the Postoperative Questionnaire which we developed for this study. (DOCX 15 kb)

## Data Availability

The datasets used and analysed during the current study are available from the corresponding author on reasonable request.

## Abbreviations

ASA: American Society of Anesthesiologists
CFNB: Continuous femoral nerve block
PACU: Post anesthesia care unit
PCA: Patient-controlled analgesia
TKA: Total knee arthroplasty
VAS: Visual Analogue Scale
