# Supplementary material for: Enhanced preoperative education about continuous femoral nerve block with patient-controlled analgesia improves the analgesic effect for patients undergoing total knee arthroplasty and reduces the workload for ward nurses
Source: BMC Anesthesiol. 2019 Aug 14;19:150. doi: 10.1186/s12871-019-0826-3 (PMC6693176; doi:10.1186/s12871-019-0826-3)
Supplement: Supplementary file 1 — This file shows the Postoperative Questionnaire which we developed for this study. (DOCX 15 kb) [file 12871_2019_826_MOESM1_ESM.docx]

**Evaluation of the effectiveness of preoperative evaluation on patient-controlled analgesia (PCA) in patients undergoing continuous femoral nerve block (CFNB)**

| **No.** | **Questions** | **T/F** |
| --- | --- | --- |
| 1 | Drug can be delivered by pressing the depressed circle of the PCA valve. |  |
| 2 | The PCA pump can continuously infuse drugs around the femoral nerve to block the nerve; patients usually do not need to press the PCA valve unless there is a palpable sensation of pain. |  |
| 3 | The interval between two successive presses of the PCA valve shall be at least 15 minutes. |  |
| 4 | The pump shall not work for more than 48 hours. |  |
| 5 | You can press the PCA valve when you experience unbearable knee pain. |  |
| 6 | You can press the PCA valve when you experience unbearable pain in the popliteal fossa. |  |
| 7 | You can press the PCA valve before and after activity/exercise. |  |
| 8 | If you feel numbness in the affected upper leg, you should ask the nurse to clamp off the pump tube and turn off the PCA pump. |  |
| 9 | After the PCA pump is withdrawn, the analgesic effect disappears after fewer than two hours. |  |
| 10 | During the use of a PCA pump, you should be extremely careful when leaving the bed, and any precautions (including holding the bed rails) should be taken to avoid falls. |  |
